# Redetermination of the crystal structure of *catena*-poly[[[bis­(ethyl­enedi­amine)­platinum(II)]-μ-iodido-[bis­(ethyl­enedi­amine)­platinum(IV)]-μ-iodido] tetra­kis­(octane-1-sulfonate) dihydrate]

**DOI:** 10.1107/S2056989015016801

**Published:** 2015-09-12

**Authors:** Nobuyuki Matsushita

**Affiliations:** a Department of Chemistry and Research Center for Smart Molecules, Rikkyo University, Nishi-Ikebukuro 3-34-1, Toshima-ku, 171-8501 Tokyo, Japan

**Keywords:** crystal structure, redetermination, *MX*-chain structure, Pt^II^/Pt^IV^ mixed-valence

## Abstract

The structure of the title compound, which represents a mixed-valence platinum(II,IV) complex, {[Pt^II^Pt^IV^I_2_(C_2_H_8_N_2_)_4_][CH_3_(CH_2_)_7_SO_3_]_4_·2H_2_O}_*n*_, has been redetermined in a different space group. In contrast to the previously reported determination in the space group *P*2_1_ *cn* [Matsushita & Taira (1999 ▸). *Synth. Met.* **102**, 1787–1788], the current model was refined in the centrosymmetric space group *Pmcn* using the original diffraction data. The title compound has a linear chain structure composed of square-planar [Pt(en)_2_]^2+^ and elongated octa­hedral *trans*-[PtI_2_(en)_2_]^2+^ cations (en is ethyl­enedi­amine) stacked alternately, bridged by the I atoms, parallel to the *c* axis. Inorganic layers aligned parallel to the *bc* plane, composed of the Pt-complex columns, the –SO_3_^−^ part of the octane-1-sulfonate anion, and the water mol­ecule of crystallization, are stacked alternately with organic layers composed of the long-chain alkyl groups along the *a* axis. The Pt and I sites are located on the same mirror plane whereby the I site is equally disordered over two positions. The Pt and I atoms form a slight zigzag ⋯I—Pt^IV^—I⋯Pt^II^⋯ chain, with Pt^IV^—I bond lengths of 2.6888 (17) and 2.7239 (17) Å, Pt^II^⋯I contacts of 3.2065 (17) and 3.1732 (16) Å, and Pt^IV^—I⋯Pt^II^ angles of 178.3 (3) and 176.7 (2)°. The mixed-valence state of the Pt site is expressed by the structural parameter δ = (Pt^IV^—I)/(Pt^II^⋯I), with values of 0.839 and 0.858 for the two independent I atoms. In the crystal, N—H⋯O hydrogen bonds involving the cationic chains, the sulfonate groups and water mol­ecules of crystallization, stabilize the columnar structure.

## Abstract 2

The redetermination of the structure of the title compound with the original measurement data revealed a centrosymmetric model in space group *Pmcn*, in contrast to the previous model in space group *P*2_1_ *cn*.

## Chemical context   

The title compound, [Pt(en)_2_][PtI_2_(en)_2_](CH_3_(CH_2_)_7_SO_3_)_4_·2H_2_O (en is ethyl­enedi­amine, C_2_N_2_H_8_), (I), is a member of the family of one-dimensional halogen-bridged mixed-valence metal complexes, formulated as [*M* ^II^(*AA*)_2_][*M* ^IV^ *X*_2_(*AA*)_2_]*Y*_4_ [*M* ^II^/*M* ^IV^ = Pt^II^/Pt^IV^, Pd^II^/Pd^IV^, Ni^II^/Ni^IV^, Pd^II^/Pt^IV^, Ni^II^/Pt^IV^; *X* = Cl, Br, I; *AA* = NH_2_(CH_2_)_2_NH_2_, *etc*; *Y* = ClO_4_^−^, HSO_4_^−^, *X* ^−^, *etc*], hereafter abbreviated as *MX*-chain compounds, which are typical mixed-valence compounds belonging to class II in the classification of Robin & Day (1967 ▸), as described in previous reports (Matsushita *et al.*, 1989 ▸, 1995 ▸; Matsushita, 1993 ▸).

The metal–halogen distances in crystals of *MX*-chain compounds characterize the physical properties based on the mixed-valence state. Compound (I) is one of the first examples of *MX*-chain structures including a long-chain alkyl group as an organic part. In a previous article (Matsushita & Taira, 1999 ▸), we have briefly reported the crystal data of (I), *i.e.* lattice parameter, space group, reliability indices, and have presented a view of the crystal packing; atomic coordinates and further structure data were not deposited at that time. The reported structure was originally refined in the non-centrosymmetric space group *P*2_1_ *cn*. However, close examination of the atomic coordinates strongly suggests that the crystal packing has an inversion center at (1/4, 1/2, 1/2). Therefore, the structure of (I) was redetermined in the centrosymmetric space group *Pmcn* and is reported here.
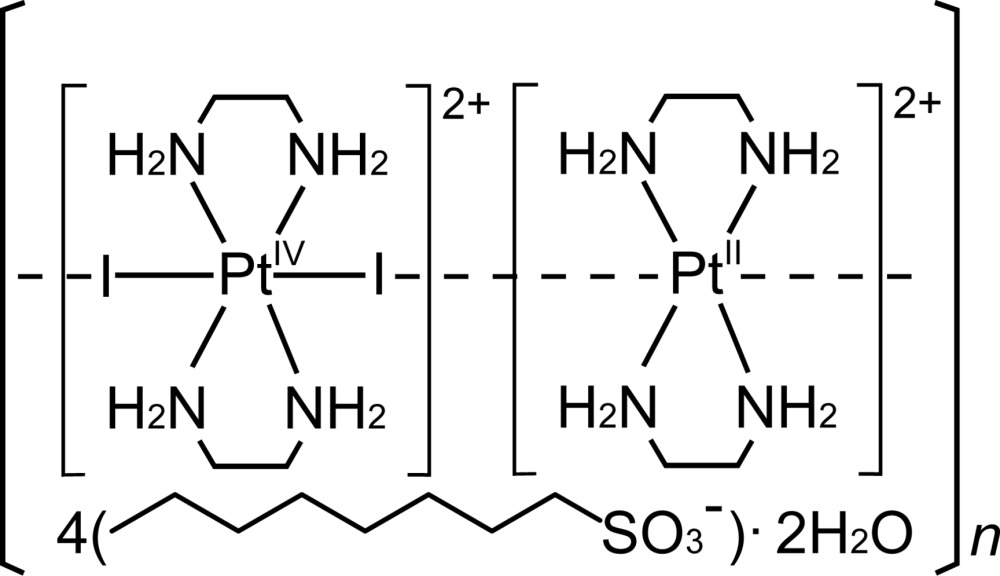


## Structural comments   

**Figure 1:**
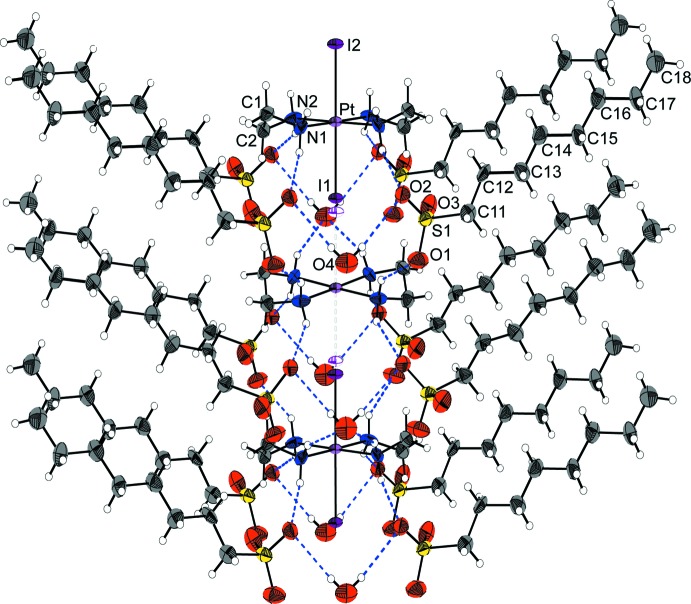
A view of the columnar structure of the title compound, showing the atomic numbering scheme. Displacement ellipsoids are drawn at the 40% probability level for non-H atoms. The violet-line ellipsoids and dashed-line bonds represent the disordered part of the Pt—I chain. Blue dashed lines represent the hydrogen bonds.

As shown in Fig. 1 ▸, the structure of (I) is built up of columns composed of square-planar [Pt(en)_2_]^2+^ and elongated octa­hedral *trans*-[PtI_2_(en)_2_]^2+^ cations stacked alternately, bridged by the I atoms, parallel to the *c* axis. The Pt and I sites lie on the same mirror plane, and form an infinite slight zigzag ⋯I—Pt^IV^—I⋯Pt^II^⋯ chain. The I atoms are not located at the exact midpoint between adjacent Pt atoms and are equally disordered over two sites close to the midpoint. Thus, the Pt site is occupationally disordered by Pt^II^ and Pt^IV^ atoms. The valence ordering of the Pt site in (I) belongs to one of three different classes of the order–disorder problem pointed out by Keller (1982 ▸). The structure of (I) can be regarded as being of the one-dimensionally ordered structure type, with the other two directions being in a disordered state. The structural order–disorder situation of the Pt site in (I) has also been observed in a number of other *MX*-chain compounds (Beauchamp *et al.*, 1982 ▸; Yamashita *et al.*, 1985 ▸; Toriumi *et al.*, 1993 ▸; Matsushita *et al.*, 1992 ▸; Huckett *et al.*, 1993 ▸; Matsushita, 2003 ▸, 2006 ▸).

With respect to the two sites for the disordered I atoms, the shorter Pt—I distances are assigned to Pt^IV^—I and the longer ones to Pt^II^⋯I, as follows: I—Pt^IV^—I; Pt—I1 = 2.6888 (17), Pt—I2 = 2.7239 (17) Å, and I1—Pt^IV^–I2 = 179.1 (3)°. I⋯Pt^II^⋯I; Pt⋯I1 = 3.2065 (17), Pt⋯I2 = 3.1732 (16) Å, and I1⋯Pt^II^⋯I2 = 177.5 (2). Bond angles of the Pt—I chain are Pt—I1⋯Pt = 178.3 (3) and Pt—I2⋯Pt = 176.7 (2)°. Other bond lengths and angles are given in Table 1 ▸.

The structural parameters indicating the mixed-valence state of the Pt atom, expressed by δ = (Pt^IV^–I)/(Pt^II^⋯I), are 0.839 and 0.858 for I1 and I2, respectively. These values are smaller than those of [Pt(pn)_2_][PtI_2_(pn)_2_](ClO_4_)_4_ (pn is 1,2-di­amino­propane) (0.937; Breer *et al.*, 1978 ▸); [Pt(pn)_2_][PtI_2_(pn)_2_]I_4_ (0.940; Endres *et al.*, 1980 ▸); [Pt(tn)_2_][PtI_2_(tn)_2_](ClO_4_)_4_ (tn is 1,3-di­amino­propane) (0.95; Cannas *et al.*, 1984 ▸); [Pt(en)_2_][PtI_2_(en)_2_](ClO_4_)_4_ (0.919; Endres *et al.*, 1979 ▸), comparable with that of [Pt(NH_3_)_4_][PtI_2_(NH_3_)_4_](HSO_4_)_4_·2H_2_O (0.834; Tanaka *et al.*, 1986 ▸), and somewhat larger than that of [Pt(en)_2_][PtI_2_(en)_2_](HPO_4_)(H_2_PO_4_)I·3H_2_O (0.812 and 0.818; Matsushita, 2006 ▸).

## Supra­molecular features   

Table 2 ▸ lists the N—H⋯O hydrogen bonds which stabilize the columnar structure composed only of cationic complexes, as shown in Fig. 1 ▸. A [Pt^II/IV^(en)_2_] unit is bound to an adjacent Pt-complex unit in the column by the hydrogen-bond linkages, NH⋯counter-anion/(water mol­ecule)⋯HN. The hydrogen-bond linkages are a common structural characteristics of *MX*-chain compounds.

**Figure 2:**
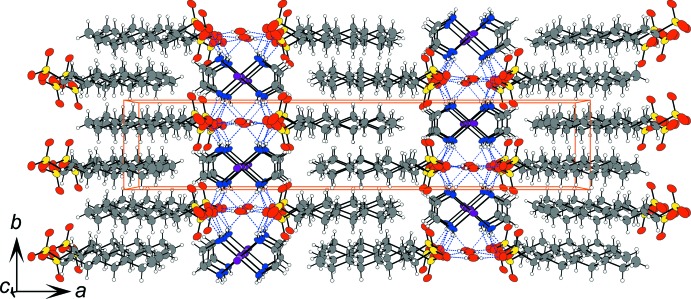
The crystal packing of the title compound, viewed along the *c* axis. Blue dashed lines represent the hydrogen bonds. Orange solid lines indicate the unit cell.

As a result of the inter­columnar hydrogen-bond linkages, as shown in Figs. 2 ▸ and 3 ▸, the columns form in layers parallel to the *bc* plane. The inorganic layer composed of the Pt-complex columns, –SO_3_^−^ part of the octane-1-sulfonate ion and the water mol­ecule of crystallization, are stacked alternately with organic layers composed of the long-chain alkyl groups along the direction of the *a* axis. The layer of the long-alkyl chain adopts an inter­digitating structure.

## Synthesis and crystallization   

The title compound was prepared by a procedure previously reported (Matsushita & Taira, 1999 ▸). Metallic bronze plate-like crystals were obtained by recrystallization from an aqueous solution on slow evaporation.

## Refinement   

Although the refinement was performed on *F* in the previous report (Matsushita & Taira, 1999 ▸), the present refinement on basis of the original diffraction data was performed on *F* ^2^. For better comparison with the previous model in space group *P*2_1_ *cn*, the non-standard setting *Pmcn* of space group No. 62 (standard setting *Pnma*) was chosen. The present model converged with improved reliability factors, and the s.u. values for the bond lengths and angles also decreased.

The arrangements of both the Pt-complex cations and the anions with the long-alkyl chain suggest that the repeat unit is half of the *c-*axis dimension. However, the different orientations of the cations and the anions cause the repeat unit to be the *c* axis. Therefore, reflections with an index of *l* = odd are very weak. As the result, a rather low percentage of reflections with [*I* > 2σ(*I*)] are observed.

The H atoms were placed in geometrically calculated positions and refined as riding (C—H = 0.97 Å and N—H = 0.90 Å), with the constraint *U*_iso_(H) = 1.5*U*_eq_(C, N). The H atoms of the water mol­ecule were located from a Fourier map and restrained with a distance of O—H = 0.82 (2) Å and *U*_iso_(H) = 1.5*U*_eq_(O). The maximum and minimum electron-density peaks lie within 0.75 Å of the Pt atom.

Crystal data, data collection and structure refinement details are summarized in Table 3 ▸.

## Supplementary Material

Crystal structure: contains datablock(s) global, I. DOI: 10.1107/S2056989015016801/wm5192sup1.cif

Structure factors: contains datablock(s) I. DOI: 10.1107/S2056989015016801/wm5192Isup2.hkl

CCDC reference: 1423010

Additional supporting information:  crystallographic information; 3D view; checkCIF report

## Figures and Tables

**Figure 3:**
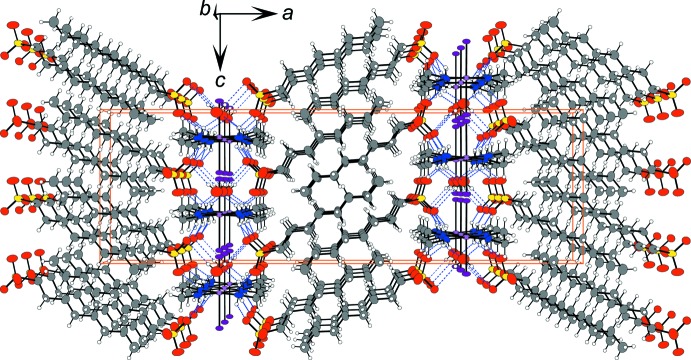
The crystal packing of the title compound viewed along the *b* axis. Blue dashed lines represent the hydrogen bonds. Orange solid lines indicate the unit cell.

**Table 1:** Selected geometric parameters (, )

PtN1	2.052(8)	PtN2	2.052(8)
			
N1^i^PtN1	96.8(5)	N1PtI1	92.1(2)
N1^i^PtN2	179.4(4)	N2PtI1	88.3(3)
N1PtN2	82.7(2)	N1PtI2^ii^	87.3(2)
N2^i^PtN2	97.7(5)	N2PtI2^ii^	92.2(3)

Symmetry codes: (i) 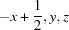; (ii) 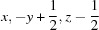.

**Table 2:** Hydrogen-bond geometry (, )

*D*H*A*	*D*H	H*A*	*D* *A*	*D*H*A*
N1H1*A*O2^iii^	0.90	2.16	2.983(11)	152
N1H1*B*O4^iv^	0.90	2.27	3.103(11)	154
N2H2*A*O1^v^	0.90	2.21	2.975(10)	142
N2H2*B*O2^vi^	0.90	2.19	2.971(11)	145
O4H4O1^v^	0.83(2)	2.23(3)	2.853(8)	132(4)
O4H4O2^v^	0.83(2)	2.44(7)	3.175(9)	148(11)

Symmetry codes: (iii) 
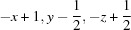
; (iv) 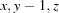; (v) 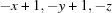; (vi) 
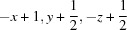
.

**Table 3:** Experimental details

Crystal data	
Chemical formula	[Pt(C_2_H_8_N_2_)_4_][PtI_2_(C_2_H_8_N_2_)_4_](C_8_H_17_SO_3_)_4_2H_2_O
*M*_r_	1693.53
Crystal system, space group	Orthorhombic, *P* *m* *c* *n*
Temperature (K)	301
*a*, *b*, *c* ()	36.997(3), 7.118(2), 11.788(3)
*V* (^3^)	3104.3(11)
*Z*	2
Radiation type	Mo *K*
(mm^1^)	5.69
Crystal size (mm)	0.17 0.15 0.05

Data collection	
Diffractometer	Rigaku AFC-5S
Absorption correction	Gaussian (Coppens *et al.*, 1965 ▸)
*T*_min_, *T*_max_	0.467, 0.757
No. of measured, independent and observed [*I* > 2(*I*)] reflections	5883, 5688, 2039
*R*_int_	0.006
(sin /)_max_ (^1^)	0.756

Refinement	
*R*[*F* ^2^ > 2(*F* ^2^)], *wR*(*F* ^2^), *S*	0.044, 0.112, 0.92
No. of reflections	5688
No. of parameters	174
No. of restraints	2
H-atom treatment	H atoms treated by a mixture of independent and constrained refinement
_max_, _min_ (e ^3^)	1.66, 2.12

Computer programs: *MSC/AFC Diffractometer Control Software* (Molecular Structure Corporation, 1988 ▸), local program *F2-AFC* (Matsushita, 1998 ▸), *SHELXS97* and *SHELXL97* (Sheldrick, 2008 ▸) and *DIAMOND* (Brandenburg, 2006 ▸).

## supplementary crystallographic information

### Crystal data

**Table:**

[Pt_2_I_2_(C_2_H_8_N_2_)_4_](C_8_H_17_SO_3_)_4_·2H_2_O	*F*(000) = 1676
*M**_r_* = 1693.53	*D*_x_ = 1.812 Mg m^−^^3^
Orthorhombic, *P**m**c**n*	Mo *K*α radiation, λ = 0.71069 Å
Hall symbol: -P 2n 2a	Cell parameters from 25 reflections
*a* = 36.997 (3) Å	θ = 10.0–14.0°
*b* = 7.118 (2) Å	µ = 5.69 mm^−^^1^
*c* = 11.788 (3) Å	*T* = 301 K
*V* = 3104.3 (11) Å^3^	Plate, metallic bronze
*Z* = 2	0.17 × 0.15 × 0.05 mm

### Data collection

**Table:**

Rigaku AFC-5S diffractometer	2039 reflections with *I* > 2σ(*I*)
Radiation source: X-ray sealed tube	*R*_int_ = 0.006
Graphite monochromator	θ_max_ = 32.5°, θ_min_ = 2.9°
ω scans	*h* = 0→55
Absorption correction: gaussian (Coppens *et al.*, 1965)	*k* = 0→10
*T*_min_ = 0.467, *T*_max_ = 0.757	*l* = 0→17
5883 measured reflections	3 standard reflections every 100 reflections
5688 independent reflections	intensity decay: none

### Refinement

**Table:**

Refinement on *F*^2^	Secondary atom site location: difference Fourier map
Least-squares matrix: full	Hydrogen site location: inferred from neighbouring sites
*R*[*F*^2^ > 2σ(*F*^2^)] = 0.044	H atoms treated by a mixture of independent and constrained refinement
*wR*(*F*^2^) = 0.112	*w* = 1/[σ^2^(*F*_o_^2^) + (0.0247*P*)^2^] where *P* = (*F*_o_^2^ + 2*F*_c_^2^)/3
*S* = 0.92	(Δ/σ)_max_ < 0.001
5688 reflections	Δρ_max_ = 1.66 e Å^−^^3^
174 parameters	Δρ_min_ = −2.12 e Å^−^^3^
2 restraints	Extinction correction: *SHELXL97* (Sheldrick, 2008), Fc^*^=kFc[1+0.001xFc^2^λ^3^/sin(2θ)]^-1/4^
Primary atom site location: heavy-atom method	Extinction coefficient: 0.00020 (3)

### Special details

**Table:**

Geometry. All e.s.d.'s (except the e.s.d. in the dihedral angle between two l.s. planes) are estimated using the full covariance matrix. The cell e.s.d.'s are taken into account individually in the estimation of e.s.d.'s in distances, angles and torsion angles; correlations between e.s.d.'s in cell parameters are only used when they are defined by crystal symmetry. An approximate (isotropic) treatment of cell e.s.d.'s is used for estimating e.s.d.'s involving l.s. planes.
Refinement. Refinement of *F*^2^ against ALL reflections. The weighted *R*-factor *wR* and goodness of fit *S* are based on *F*^2^, conventional *R*-factors *R* are based on *F*, with *F* set to zero for negative *F*^2^. The threshold expression of *F*^2^ > 2σ(*F*^2^) is used only for calculating *R*-factors(gt) *etc*. and is not relevant to the choice of reflections for refinement. *R*-factors based on *F*^2^ are statistically about twice as large as those based on *F*, and *R*- factors based on ALL data will be even larger.

### Fractional atomic coordinates and isotropic or equivalent isotropic displacement parameters (Å^2^)

**Table:**

	*x*	*y*	*z*	*U*_iso_*/*U*_eq_	Occ. (<1)
Pt	0.2500	0.25626 (11)	0.18219 (3)	0.02517 (11)	
I1	0.2500	0.2445 (9)	0.41018 (13)	0.0337 (5)	0.50
I2	0.2500	0.2378 (9)	0.45114 (13)	0.0344 (6)	0.50
N1	0.2915 (2)	0.0656 (11)	0.1722 (6)	0.036 (2)	
H1A	0.2941	0.0071	0.2394	0.053*	
H1B	0.2862	−0.0217	0.1194	0.053*	
N2	0.2918 (3)	0.4454 (12)	0.1908 (7)	0.043 (3)	
H2A	0.2934	0.5098	0.1253	0.064*	
H2B	0.2878	0.5277	0.2474	0.064*	
C1	0.3255 (3)	0.1620 (14)	0.1418 (8)	0.050 (3)	
H1C	0.3260	0.1911	0.0614	0.075*	
H1D	0.3462	0.0836	0.1598	0.075*	
C2	0.3261 (3)	0.3405 (14)	0.2118 (8)	0.048 (3)	
H2C	0.3283	0.3100	0.2917	0.072*	
H2D	0.3467	0.4173	0.1903	0.072*	
O4	0.2500	0.7653 (16)	0.0327 (9)	0.073 (3)	
H4	0.2677 (5)	0.718 (13)	0.000 (7)	0.109*	
S1	0.66363 (7)	0.3288 (3)	0.07740 (19)	0.0466 (6)	
O1	0.6733 (2)	0.2765 (13)	−0.0376 (5)	0.080 (2)	
O2	0.69019 (16)	0.2676 (13)	0.1594 (4)	0.0525 (16)	
O3	0.65542 (19)	0.5260 (9)	0.0862 (6)	0.068 (2)	
C11	0.6225 (3)	0.2054 (12)	0.1073 (7)	0.053 (3)	
H11A	0.6059	0.2242	0.0448	0.079*	
H11B	0.6277	0.0721	0.1119	0.079*	
C12	0.6044 (2)	0.2663 (17)	0.2159 (7)	0.048 (2)	
H12A	0.6020	0.4020	0.2165	0.072*	
H12B	0.6192	0.2303	0.2801	0.072*	
C13	0.5670 (3)	0.1764 (14)	0.2275 (8)	0.058 (3)	
H13A	0.5534	0.1998	0.1585	0.087*	
H13B	0.5699	0.0416	0.2349	0.087*	
C14	0.5457 (2)	0.246 (2)	0.3255 (7)	0.056 (2)	
H14A	0.5445	0.3824	0.3212	0.084*	
H14B	0.5585	0.2144	0.3947	0.084*	
C15	0.5077 (3)	0.1697 (15)	0.3330 (7)	0.056 (3)	
H15A	0.5091	0.0344	0.3419	0.084*	
H15B	0.4955	0.1945	0.2617	0.084*	
C16	0.4847 (2)	0.2488 (16)	0.4288 (7)	0.058 (2)	
H16A	0.4968	0.2239	0.5002	0.087*	
H16B	0.4832	0.3840	0.4198	0.087*	
C17	0.4468 (3)	0.1704 (13)	0.4350 (9)	0.060 (3)	
H17A	0.4344	0.1988	0.3645	0.090*	
H17B	0.4483	0.0347	0.4414	0.090*	
C18	0.4244 (3)	0.244 (2)	0.5322 (8)	0.078 (3)	
H18A	0.4356	0.2109	0.6028	0.117*	
H18B	0.4006	0.1902	0.5290	0.117*	
H18C	0.4226	0.3784	0.5268	0.117*	

### Atomic displacement parameters (Å^2^)

**Table:**

	*U*^11^	*U*^22^	*U*^33^	*U*^12^	*U*^13^	*U*^23^
Pt	0.0375 (2)	0.02157 (17)	0.01644 (15)	0.000	0.000	0.0004 (3)
I1	0.0448 (11)	0.0391 (9)	0.0173 (7)	0.000	0.000	0.002 (2)
I2	0.0552 (12)	0.0308 (11)	0.0173 (8)	0.000	0.000	−0.0001 (18)
N1	0.035 (6)	0.036 (5)	0.036 (5)	0.007 (4)	0.012 (4)	0.000 (3)
N2	0.068 (9)	0.034 (5)	0.026 (4)	−0.020 (5)	0.006 (4)	0.003 (3)
C1	0.044 (7)	0.069 (7)	0.037 (5)	0.015 (5)	0.010 (5)	0.002 (5)
C2	0.041 (6)	0.055 (6)	0.048 (5)	−0.009 (5)	−0.005 (5)	0.013 (5)
O4	0.100 (8)	0.036 (5)	0.081 (7)	0.000	0.000	−0.023 (6)
S1	0.0548 (15)	0.0516 (13)	0.0334 (11)	−0.0170 (11)	−0.0039 (12)	0.0021 (10)
O1	0.099 (6)	0.099 (6)	0.041 (3)	−0.040 (6)	0.009 (4)	0.002 (5)
O2	0.048 (3)	0.064 (4)	0.045 (3)	−0.009 (5)	−0.004 (3)	0.008 (4)
O3	0.064 (5)	0.048 (4)	0.091 (5)	−0.019 (3)	−0.016 (4)	0.014 (4)
C11	0.058 (6)	0.050 (6)	0.049 (5)	−0.017 (5)	−0.003 (5)	0.012 (4)
C12	0.045 (5)	0.052 (6)	0.048 (4)	−0.023 (6)	−0.019 (4)	0.003 (6)
C13	0.055 (7)	0.062 (6)	0.057 (6)	−0.010 (5)	0.007 (6)	0.012 (5)
C14	0.056 (5)	0.055 (5)	0.058 (5)	−0.022 (8)	−0.007 (5)	0.000 (7)
C15	0.042 (6)	0.063 (6)	0.064 (7)	−0.014 (5)	0.006 (5)	0.017 (5)
C16	0.053 (5)	0.056 (5)	0.064 (5)	−0.017 (7)	−0.005 (5)	0.021 (9)
C17	0.045 (6)	0.054 (5)	0.081 (7)	−0.008 (5)	0.014 (7)	0.015 (6)
C18	0.066 (7)	0.086 (7)	0.083 (7)	−0.004 (10)	0.005 (6)	−0.009 (10)

### Geometric parameters (Å, º)

**Table:**

Pt—N1^i^	2.052 (8)	C11—C12	1.509 (11)
Pt—N1	2.052 (8)	C11—H11A	0.9700
Pt—N2^i^	2.052 (8)	C11—H11B	0.9700
Pt—N2	2.052 (8)	C12—C13	1.531 (12)
Pt—I1	2.6888 (17)	C12—H12A	0.9700
Pt—I2^ii^	2.7239 (17)	C12—H12B	0.9700
Pt—I2	3.1732 (16)	C13—C14	1.483 (12)
Pt—I1^iii^	3.2065 (17)	C13—H13A	0.9700
N1—C1	1.479 (11)	C13—H13B	0.9700
N1—H1A	0.9000	C14—C15	1.513 (12)
N1—H1B	0.9000	C14—H14A	0.9700
N2—C2	1.495 (12)	C14—H14B	0.9700
N2—H2A	0.9000	C15—C16	1.522 (12)
N2—H2B	0.9000	C15—H15A	0.9700
C1—C2	1.515 (12)	C15—H15B	0.9700
C1—H1C	0.9700	C16—C17	1.511 (12)
C1—H1D	0.9700	C16—H16A	0.9700
C2—H2C	0.9700	C16—H16B	0.9700
C2—H2D	0.9700	C17—C18	1.510 (12)
O4—H4	0.83 (2)	C17—H17A	0.9700
S1—O3	1.440 (7)	C17—H17B	0.9700
S1—O2	1.446 (6)	C18—H18A	0.9600
S1—O1	1.451 (7)	C18—H18B	0.9600
S1—C11	1.791 (9)	C18—H18C	0.9600
			
N1^i^—Pt—N1	96.8 (5)	O3—S1—O2	112.9 (5)
N1^i^—Pt—N2^i^	82.7 (2)	O3—S1—O1	111.7 (5)
N1—Pt—N2^i^	179.4 (4)	O2—S1—O1	112.3 (4)
N1^i^—Pt—N2	179.4 (4)	O3—S1—C11	106.5 (5)
N1—Pt—N2	82.7 (2)	O2—S1—C11	107.3 (4)
N2^i^—Pt—N2	97.7 (5)	O1—S1—C11	105.5 (4)
N1^i^—Pt—I1	92.1 (2)	C12—C11—S1	113.8 (6)
N1—Pt—I1	92.1 (2)	C12—C11—H11A	108.8
N2^i^—Pt—I1	88.3 (3)	S1—C11—H11A	108.8
N2—Pt—I1	88.3 (3)	C12—C11—H11B	108.8
N1^i^—Pt—I2^ii^	87.3 (2)	S1—C11—H11B	108.8
N1—Pt—I2^ii^	87.3 (2)	H11A—C11—H11B	107.7
N2^i^—Pt—I2^ii^	92.2 (3)	C11—C12—C13	111.0 (8)
N2—Pt—I2^ii^	92.2 (3)	C11—C12—H12A	109.4
I1—Pt—I2^ii^	179.1 (3)	C13—C12—H12A	109.4
N1^i^—Pt—I2	91.7 (2)	C11—C12—H12B	109.4
N1—Pt—I2	91.7 (2)	C13—C12—H12B	109.4
N2^i^—Pt—I2	88.7 (2)	H12A—C12—H12B	108.0
N2—Pt—I2	88.7 (2)	C14—C13—C12	114.1 (8)
I1—Pt—I2	0.6 (2)	C14—C13—H13A	108.7
I2^ii^—Pt—I2	178.52 (4)	C12—C13—H13A	108.7
N1^i^—Pt—I1^iii^	86.6 (2)	C14—C13—H13B	108.7
N1—Pt—I1^iii^	86.6 (2)	C12—C13—H13B	108.7
N2^i^—Pt—I1^iii^	92.9 (2)	H13A—C13—H13B	107.6
N2—Pt—I1^iii^	92.9 (2)	C13—C14—C15	114.6 (9)
I1—Pt—I1^iii^	178.12 (4)	C13—C14—H14A	108.6
I2^ii^—Pt—I1^iii^	1.0 (2)	C15—C14—H14A	108.6
I2—Pt—I1^iii^	177.5 (2)	C13—C14—H14B	108.6
Pt—I1—Pt^iv^	178.3 (3)	C15—C14—H14B	108.6
Pt^iv^—I2—Pt	176.7 (2)	H14A—C14—H14B	107.6
C1—N1—Pt	110.1 (6)	C14—C15—C16	115.5 (8)
C1—N1—H1A	109.6	C14—C15—H15A	108.4
Pt—N1—H1A	109.6	C16—C15—H15A	108.4
C1—N1—H1B	109.6	C14—C15—H15B	108.4
Pt—N1—H1B	109.6	C16—C15—H15B	108.4
H1A—N1—H1B	108.1	H15A—C15—H15B	107.5
C2—N2—Pt	108.7 (6)	C17—C16—C15	114.7 (9)
C2—N2—H2A	109.9	C17—C16—H16A	108.6
Pt—N2—H2A	109.9	C15—C16—H16A	108.6
C2—N2—H2B	109.9	C17—C16—H16B	108.6
Pt—N2—H2B	109.9	C15—C16—H16B	108.6
H2A—N2—H2B	108.3	H16A—C16—H16B	107.6
N1—C1—C2	105.7 (8)	C18—C17—C16	114.7 (9)
N1—C1—H1C	110.6	C18—C17—H17A	108.6
C2—C1—H1C	110.6	C16—C17—H17A	108.6
N1—C1—H1D	110.6	C18—C17—H17B	108.6
C2—C1—H1D	110.6	C16—C17—H17B	108.6
H1C—C1—H1D	108.7	H17A—C17—H17B	107.6
N2—C2—C1	108.4 (8)	C17—C18—H18A	109.5
N2—C2—H2C	110.0	C17—C18—H18B	109.5
C1—C2—H2C	110.0	H18A—C18—H18B	109.5
N2—C2—H2D	110.0	C17—C18—H18C	109.5
C1—C2—H2D	110.0	H18A—C18—H18C	109.5
H2C—C2—H2D	108.4	H18B—C18—H18C	109.5

Symmetry codes: (i) −*x*+1/2, *y*, *z*; (ii) *x*, −*y*+1/2, *z*−1/2; (iii) −*x*+1/2, −*y*+1/2, *z*−1/2; (iv) *x*, −*y*+1/2, *z*+1/2.

### Hydrogen-bond geometry (Å, º)

**Table:**

*D*—H···*A*	*D*—H	H···*A*	*D*···*A*	*D*—H···*A*
N1—H1*A*···O2^v^	0.90	2.16	2.983 (11)	152
N1—H1*B*···O4^vi^	0.90	2.27	3.103 (11)	154
N2—H2*A*···O1^vii^	0.90	2.21	2.975 (10)	142
N2—H2*B*···O2^viii^	0.90	2.19	2.971 (11)	145
O4—H4···O1^vii^	0.83 (2)	2.23 (3)	2.853 (8)	132 (4)
O4—H4···O2^vii^	0.83 (2)	2.44 (7)	3.175 (9)	148 (11)

Symmetry codes: (v) −*x*+1, *y*−1/2, −*z*+1/2; (vi) *x*, *y*−1, *z*; (vii) −*x*+1, −*y*+1, −*z*; (viii) −*x*+1, *y*+1/2, −*z*+1/2.
